# Olfactory experience shapes the evaluation of odour similarity in ants: a behavioural and computational analysis

**DOI:** 10.1098/rspb.2016.0551

**Published:** 2016-08-31

**Authors:** Margot Perez, Thomas Nowotny, Patrizia d'Ettorre, Martin Giurfa

**Affiliations:** 1 Laboratory of Experimental and Comparative Ethology (LEEC), University Paris 13, Sorbonne Paris Cité, Villetaneuse, France; 2 Centre National de la Recherche Scientifique (CNRS), Research Centre on Animal Cognition (UMR5169), Toulouse, France; 3 Research Centre on Animal Cognition (UMR5169), University Paul-Sabatier, Toulouse, France; 4 Centre for Computational Neuroscience and Robotics, School of Engineering and Informatics, University of Sussex, Brighton, UK

**Keywords:** olfactory learning, olfactory perception, generalization, generalization gradient, perceptual modelling, ant

## Abstract

Perceptual similarity between stimuli is often assessed via generalization, the response to stimuli that are similar to the one which was previously conditioned. Although conditioning procedures are variable, studies on how this variation may affect perceptual similarity remain scarce. Here, we use a combination of behavioural and computational analyses to investigate the influence of olfactory conditioning procedures on odour generalization in ants. Insects were trained following either absolute conditioning, in which a single odour (an aldehyde) was rewarded with sucrose, or differential conditioning, in which one odour (the same aldehyde) was similarly rewarded and another odour (an aldehyde differing in carbon-chain length) was punished with quinine. The response to the trained odours and generalization to other aldehydes were assessed. We show that olfactory similarity, rather than being immutable, varies with the conditioning procedure. Compared with absolute conditioning, differential conditioning enhances olfactory discrimination. This improvement is best described by a multiplicative interaction between two independent processes, the excitatory and inhibitory generalization gradients induced by the rewarded and the punished odour, respectively. We show that olfactory similarity is dramatically shaped by an individual's perceptual experience and suggest a new hypothesis for the nature of stimulus interactions underlying experience-dependent changes in perceptual similarity.

## 1. Introduction

The capacity to associate stimuli with different outcomes and to differentiate these stimuli from similar but irrelevant ones is essential for many behaviours such as feeding, mating or communicating. Discrimination abilities (i.e. treating different stimuli as distinct) displayed by most living animals are, therefore, critical for survival and reproductive success. However, in an environment that is continuously changing, responding differently to stimuli that differ only slightly is not necessarily advantageous. For instance, the quantity or quality of volatiles emitted by a flower can vary in time and space [1] without necessarily signifying a difference in nectar quality. As similar stimuli often share comparable outcomes, most living animals display the capacity to generalize, i.e. to treat different but similar stimuli as equivalent [2–4], an efficient strategy to cope with stimulus variations or stimulus novelty.

The degree of generalization across stimuli is determined by their degree of similarity along a given perceptual dimension [3,4]. A generalization gradient can be drawn by training animals to a particular stimulus and then testing their responses to other stimuli varying along a chosen dimension. Generalization gradients typically show stronger responses to stimuli that are similar to the trained one and a progressive decrease in responses with decreased similarity [4,5]. For instance, in the olfactory modality, carbon-chain length of odours sharing the same functional group is a relevant dimension (among others) for graded odourant similarity in many taxa (vertebrates [6–10]; invertebrates [11–14]); i.e. the smaller the difference in carbon-chain length between odours, the stronger the generalization.

Perceptual similarity may also be influenced by the perceiver's experience. Indeed, discrimination abilities between similar stimuli can be improved by differential learning, which consists of the parallel association of one stimulus with a reward and of another stimulus with an absence of reward or a punishment. The effect of conditioning procedures on the ability to shift from generalization to discrimination between similar stimuli has been shown in some species and sensory modalities such as vision and olfaction [10,15–23] but low numbers of stimuli used or a lack of systematic analyses along perceptual dimensions sometimes makes it difficult to assess how exactly experience modulates perceptual similarity.

Despite the extensive use of insects as behavioural and neurophysiological models for the study of olfactory perception and learning [24,25], the influence of experience on odour perception has been poorly investigated (but see [13,23]). Ants rely highly on olfactory cues in their natural environment and in social interactions [26], and represent, therefore, a suitable model for the study of olfactory perception and learning [27–32]. They increase the diversity of studies on insect olfaction, which have mostly focused on bees, moths and fruit flies. Controlled olfactory conditioning protocols have been established for ants, which allow training them under absolute or differential conditioning, i.e. with a single stimulus rewarded (the conditioned stimulus, CS+), or with one stimulus rewarded (CS+) and another stimulus non-rewarded or punished (CS−) [27–30,33–35]. Both protocols have been used to investigate olfactory learning and generalization in ants, but no comparative study has been undertaken to determine the specific influence of experimental procedures on these abilities. Here, we compared generalization gradients after either absolute or differential conditioning in the ant *Camponotus aethiops.* We show that discriminatory abilities improve after differential training, thus demonstrating that olfactory similarity relationships are not immutable but vary with experience. Computational analyses of our behavioural data indicate that the observed differences in generalization performance can be well explained by differences in the generalization gradients induced by our conditioning protocols. Our study allows, therefore, a better understanding of how an individual's experience shapes similarity relationships between stimuli.

## 2. Material and methods

### (a) Individual handling

Medium-sized foragers were collected and anaesthetized on ice for harnessing in individual holders as previously described [14,35]. Fixed ants could only move their antennae and mouthparts. They were then left in a quiet and humid place during 3 h to recover from anaesthesia and accustom to harnessing conditions (see the electronic supplementary material).

### (b) Stimuli

Four aldehydes that varied in carbon-chain length from six to nine carbons (i.e. hexanal, heptanal, octanal and nonanal; Sigma Aldrich, France) were used as conditioned and test stimuli. Before each training phase, two microliters of pure odourant were applied onto a 1 cm^2^ piece of filter paper, which was then inserted in a plastic 10 ml syringe. The rewarded odour CS+ was paired with an appetitive 50% sucrose solution (w/w); when ants were trained in differential conditioning, besides the CS+, a punished odour CS− was paired with an aversive 1% quinine solution (w/w) (purity 90%, Sigma Aldrich, France).

### (c) Conditioning and test procedures

To test whether olfactory generalization gradients are influenced by olfactory experience, ants were subjected to either absolute or differential conditioning of the *maxilla-labium* extension response (MaLER), an appetitive reaction to sucrose stimulation [34].

Four aldehydes were trained in *absolute conditioning*: hexanal+; heptanal+; octanal+; nonanal+, where ‘+’ indicates the presence of reward. In *differential conditioning*, four odourant combinations, each one presenting two odours differing in two carbons, were trained: hexanal+/octanal−; heptanal+/nonanal−; octanal+/hexanal−; nonanal+/heptanal−, where ‘−’ indicates the presence of punishment. Training consisted of 12 trials: for absolute conditioning, six CS+ and six blank trials (see the electronic supplementary material); for differential conditioning six CS+ and six CS− trials. Trials were performed in pseudo-random order, e.g. ABBABAABABBA. In both cases, ants were tested with the four aldehydes in a randomized order 15 min after the last conditioning trial (more details on conditioning and test procedures and statistical analyses in the electronic supplementary material).

### (d) Generalization-gradient modelling

We analysed the behavioural responses by fitting a simple model of Gaussian excitatory and inhibitory generalization gradients for the CS+ and the CS−, respectively. To this end, we fitted for each experiment a Gaussian function *G_σ_* with maximal amplitude 100, standard deviation *σ* and centred on the CS+, to the response percentages obtained after absolute conditioning. Simultaneously, we fitted the product *G_σ_* × (1 − *G_σ_*_′_) to the data obtained after differential conditioning, with *G_σ_*_′_ being centred on the CS−. This constituted a two-parameter fit (of *σ* and *σ*′) to eight data points (four from absolute conditioning and four from differential conditioning). The fit was performed as a least-mean-squares fit with the *lsqnonlin* function in Matlab (Natick, MA, USA).

*G_σ_* and *G_σ_*_′_ can be interpreted as probabilities (expressed in %) of response and of inhibition of response, respectively. The overall probability of responding after differential conditioning is thus the probability to respond and to not be inhibited to respond, i.e. *G_σ_* × (1 − *G_σ_*_′_). Here, the term (1 − *G_σ_*_′_) corresponds to logical negation, i.e. the event of ‘not being inhibited to respond’; the multiplication of *G_σ_* and (1 − *G_σ_*_′_) corresponds to the logical ‘and’ operation (assuming that responding and not being inhibited to respond are statistically independent). More details about the choice of the model are in the electronic supplementary material.

## 3. Results

### (a) Olfactory learning

**Figure 1.**
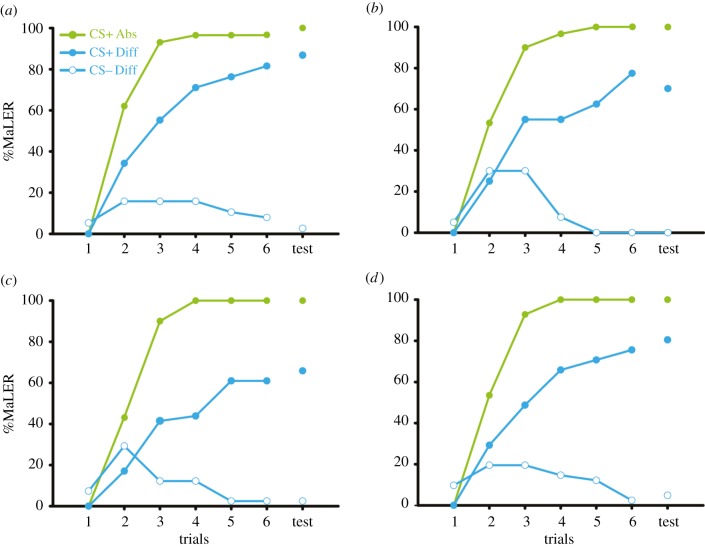
Ants trained with absolute conditioning learn faster and better than ants trained with differential conditioning. The figure shows the percentage of ants responding with the *maxilla-labium* extension response (%MaLER) to the rewarded odour after absolute conditioning (CS+, green full dots) and to the rewarded and punished odours after differential conditioning (CS+, blue full dots; CS−, blue empty dots) along the conditioning trials. The levels of response to the trained odours in the tests are shown by single dots in the same graphs. (*a*) Hexanal+ (*n* = 29) versus hexanal+/octanal− (*n* = 38); (*b*) heptanal+ (*n* = 30) versus heptanal+/nonanal− (*n* = 40); (*c*) octanal+ (*n* = 30) versus octanal+/hexanal− (*n* = 41); and (*d*) nonanal+ (*n* = 28) versus nonanal+/heptanal− (*n* = 41). All odours were successfully learnt and all odour combinations were successfully differentiated. The presence of the CS− in differential conditioning reduced the learning success of the CS+ as shown by lower rates of acquisition and lower levels of responses to the CS+ in the test, compared with ants trained with absolute conditioning.

Ants trained following an absolute conditioning protocol (green curves in figure 1) learnt successfully all four odours, as revealed by a significant effect of conditioning trials on the level of conditioned responses (22.50 < *χ*^2^ < 37.29; *p* < 0.001 for all odours), which reached approximately 100% in the last trial. All trained odours were well retained 15 min after training as shown by the test responses (green dots in figure 1), which did not differ from those recorded in the last conditioning trial (for all odours, *χ*^2^ = 0; *p* = 1).

Ants trained following a differential conditioning protocol (blue curves in figure 1) learnt to differentiate the CS+ from the CS− in all odour combinations tested, as indicated by a significant interaction between trials and CSs (39.49 < *χ*^2^ < 57.19; *p* < 0.001 in all cases) and by the high level of conditioned responses to the CS+ (approx. 74%) and the low level of response to the CS− (approx. 3%) reached in the last trial. A significant effect of trial was found for the responses to the CS+, thus indicating that ants increased their response to the rewarded odour along successive trials (51.80 < *χ*^2^ < 65.59; *p* < 0.001 for all combinations). Post hoc analyses also revealed a significant effect of trials on the CS− responses for the octanal+/hexanal− and heptanal+/nonanal− combinations (7.08 < *χ*^2^ < 9.55; *p* < 0.01 in both cases), indicating that, in these cases, ants learnt to stop responding to the CS− although the level of responses to this stimulus was relatively high in the first trials. For the other two combinations (hexanal+/octanal− and nonanal+/heptanal), the level of responses to the CS− did not change along trials (*χ*^2^ = 0; *p* = 0.95 and *χ*^2^ = 2.45; *p* = 0.10, respectively) and remained low.

Ants remembered well the rewarded and punished odours 15 min after differential conditioning as they showed, respectively, high (around 76%) and low (around 3%) levels of conditioned responses in the test to these stimuli (24.038 < McNemar's *χ*^2^ < 30.031, *p* < 0.001 in all cases; full versus empty blue dots in figure 1). For all four odour combinations, test responses were similar to those recorded in the last conditioning trial for both CSs (CS+: 0.17 < McNemar's *χ*^2^ < 0.44; 0.51 < *p* < 0.68 and CS−: 0 < McNemar's *χ*^2^ < 0.5; 0.47 < *p* < 1).

### (b) Comparing CS+ learning between conditioning protocols

A comparative analysis between absolute and differential conditioning is possible by focusing on how the same CS+ odour is learnt and memorized depending on the absence/presence of a CS− odour. Figure 1 shows that the conditioning procedure affected the acquisition rate of the CS+ as a significant interaction between trials and conditioning procedure was found in all cases (comparison of blue and green full dotted curves; 6.32 < *χ*^2^ < 13.75; *p* < 0.05). Consequently, the level of CS+ responses in the test was significantly lower for ants trained with differential conditioning, except in the case of hexanal+ versus hexanal+/octanal− (figure 1*a*) where it was close to significance (Fisher's exact test, 0.001 < *p* < 0.06). The better learning performances obtained with absolute rather than with differential conditioning indicate that the introduction of a CS− differing in two carbons from the CS+ reduced significantly the learning success of the CS+; i.e. learning to discriminate similar odours is more difficult than learning the absolute properties of a single rewarded odour.

### (c) Olfactory generalization

**Figure 2.**
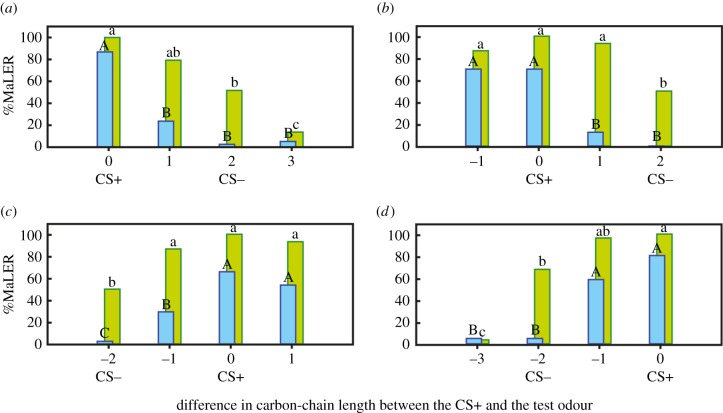
Differential conditioning improves olfactory discrimination. The %MaLER to the test odours hexanal, heptanal, octanal and nonanal (bars from left to right) is represented for ants trained with absolute (green bars) and differential (blue bars) conditioning. The *x*-axis shows the difference in carbon-chain length between the CS+ and the test odour. Odours used as CS+ and CS− during conditioning are indicated. (*a*) Hexanal+ versus hexanal+/octanal−; (*b*) heptanal+ versus heptanal+/nonanal−; (*c*) octanal+ versus octanal+/hexanal−; and (*d*) nonanal+ versus nonanal+/heptanal−. The ants' responses to the test odours were different after absolute (29.4 < Cochran's *Q* < 61.81; *p* < 0.001) and differential (51.78 < Cochran's *Q* < 75; *p* < 0.001) conditioning. Different letters indicate significant differences in the levels of responses to test odours for ants trained with absolute (lower case) and differential conditioning (capital letters; multiple McNemar's *χ*^2^-tests with sequential Bonferroni's corrections). Generalization gradients obtained after absolute conditioning (green bars) show that carbon-chain length is a relevant dimension for perceptual similarity as the response levels were inversely related to the difference in the number of carbons between the CS+ and the test odours. Generalization gradients obtained after differential conditioning (blue bars) illustrate the resulting partial improvement of discrimination as the level of response for odours differing from the CS+ in 1 (upper panels) or −1 carbon (lower panels) were significantly lower than the level of response to the CS+, whereas such difference did not exist after absolute conditioning.

After absolute conditioning, ants showed not only a high level of responses to their conditioning odour but also to the other test odours (green bars in figure 2), i.e. ants highly generalized among aldehydes. Generalization levels were inversely related to the difference in the carbon-chain length between the conditioning and the test odour: the lower the difference in the number of carbon atoms, the higher the level of generalization. For instance, after hexanal+ training (green bars in figure 2*a*), ants responded similarly to heptanal (differing in 1 carbon from hexanal), less to octanal (differing in two carbons from hexanal) and even less to nonanal (differing in three carbons from hexanal). The shape of the generalization gradients obtained after absolute conditioning shows, therefore, that carbon-chain length is a relevant dimension for graded olfactory similarity in ants.

Olfactory similarity was affected by the training procedure as revealed by differences in generalization gradients after absolute and differential conditioning (comparison of green versus blue bars in figure 2). Indeed, after differential conditioning, generalization levels were lower for odours that had a carbon-chain length relation to the CS+ similar to that of the CS−. In other words, if the CS− had a shorter carbon-chain length than that of the CS+, discrimination was improved only for odours having a shorter carbon-chain length than that of the CS+. Reciprocally, if the CS− had a longer carbon-chain length than that of the CS+, discrimination was improved for odours having a longer carbon-chain length than that of the CS+. For instance, after octanal+/hexanal− training (i.e. CS− with shorter carbon-chain length than CS+), the level of responses to heptanal, which has a shorter carbon-chain length than octanal, is significantly lower than the level of response to octanal (blue bars in figure 2*c*), whereas these levels were similar after absolute conditioning (green bars in figure 2*c*). On the other hand, the level of responses to nonanal, which has a longer carbon-chain length than octanal, was similar to that of octanal (blue bars in figure 2*c*), as found after absolute conditioning (green bars in figure 2*c*). In the case of nonanal+/heptanal− (figure 2*d*), the difference in response between nonanal and octanal was almost significant (*p*_corr_ = 0.05), thus indicating that even after differential conditioning, high levels of generalization can still be observed. In general, however, differential learning improved the discrimination abilities of ants but only for odours that had a carbon-chain length relation to the CS+ similar to that of the CS−. Consequently, generalization gradients after differential conditioning were asymmetric around the CS+, with higher levels of response in the case of odours away from the CS+ in the direction opposite to the CS−; a phenomenon called area shift [36].

The enhanced perceptual discrimination resulting from differential learning and the area shift effect are commonly accounted for by the assumption that generalization performances result from an interaction between excitatory and inhibitory generalization gradients mediated by the CS+ and the CS−, respectively [4,15,37]. We thus aimed at modelling excitatory and inhibitory generalization gradients in order to better explain the improvement of ants' discrimination abilities.

### (d) Generalization-gradient modelling

**Figure 3.**
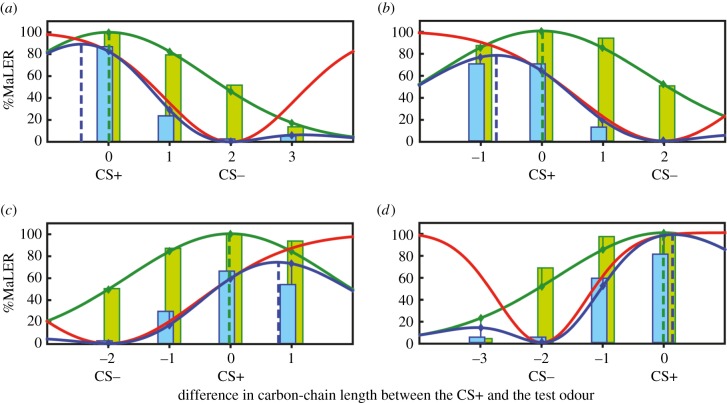
The enhancement of olfactory discrimination after differential conditioning can be explained by the interaction between excitatory and inhibitory gradients mediated by the rewarded and the punished odours, respectively. Fits of the experimental data with combinations of ‘excitatory’ and ‘inhibitory’ generalization gradients. Axis and colour code of bars as in figure 2. Excitatory Gaussian generalization gradients (green lines) were fitted to the test responses after absolute conditioning (green bars). The combination (blue lines) of the excitatory gradient and a hypothesized inhibitory generalization gradient (red lines) was fitted to the test responses after differential conditioning (blue bars). The position of each gradient's peak is indicated by vertical dotted lines in their respective colours. The peak positions of excitatory and inhibitory generalization gradients were fixed to the CS+ and CS−, respectively. (*a*) Hexanal+ versus hexanal+/octanal−; (*b*) heptanal+ versus heptanal+/nonanal−; (*c*) octanal+ versus octanal+/hexanal−; and (*d*) nonanal+ versus nonanal+/heptanal−. The interaction between excitatory and inhibitory gradients in this model was multiplicative (alternative models are shown in the electronic supplementary material, figures S1–S3). The combined gradient provided a good fit to the differential conditioning data given that there were only two free parameters (the widths of the excitatory and inhibitory generalization gradients) and the area shift is clearly visible.

Excitatory generalization gradients (green lines in figure 3) were obtained by fitting a Gaussian function to the data obtained after absolute conditioning. This function was characterized by one free parameter: the standard deviation *σ* (table 1). Hypothesized inhibitory generalization gradients (red lines in figure 3), characterized by the standard deviation *σ*′ (table 1), were obtained from the combination of excitatory generalization gradients and the level of response to the four odours after differential conditioning (blue lines in figure 3). Both excitatory and inhibitory generalization gradients were assumed to have a fixed amplitude of 100%. For excitatory generalization gradients, this assumption mirrors the experimental data obtained after absolute conditioning where all ants responded to their trained odour in the test (figures 1 and 2). The peaks of the excitatory (green dashed vertical lines) and inhibitory generalization gradients were centred on the CS+ and the CS−, respectively, as we assumed that the maximum of excitation and inhibition were provided by these stimuli. Generalization gradients after differential conditioning were only partially accounted for by additive fits of excitatory and inhibitory generalization gradients as shown by high residual errors (REs; see the electronic supplementary material), despite the fact that this kind of interaction is assumed in generalization theories [15,37,38]. However, the most convincing fits were obtained with a multiplicative fit of excitatory and inhibitory generalization gradients, as shown by the low RE across all experiments (RE = 21.29, i.e. the typical deviation of the fit from the data is RE/8 = 2.66) and by the visual inspection of the fits (see the electronic supplementary material, figures S1–S3 and table S1 for a full comparison). The success of the multiplicative fit suggests that an interpretation of excitation and inhibition as two interacting, independent probabilistic events may be more appropriate than a view of super-imposed excitatory and inhibitory ‘potentials’.

**Table 1.** Fitted parameters for excitatory and inhibitory generalization gradients. (The parameters *σ* and *σ′* denote the standard deviations of the curves corresponding to the excitatory and inhibitory generalization gradients, respectively.)

experiment	*σ* (excitatory)	*σ′* (inhibitory)
hexanal+/octanal−	1.606	1.071
heptanal+/nonanal−	1.736	1.403
octanal+/hexanal−	1.682	1.492
nonanal+/heptanal−	1.730	0.732

Figure 3 shows that differential conditioning induced a peak shift in generalization gradient. Indeed, in all cases, the position of the peak (blue dashed vertical line) was not centred on the CS+ contrary to the peak obtained after absolute conditioning, which was centred on the CS+ (green dashed vertical line). The peak of the generalization gradient after differential conditioning was shifted away from the CS+ in the direction opposite to the CS−. This peak shift mirrors the area shift found in our behavioural experiments. Moreover, excitatory and inhibitory generalization gradients differed consistently in their shapes. In all experiments, the standard deviation (*σ*′) of the inhibitory generalization gradient was lower than the standard deviation (*σ*) of the excitatory generalization gradient (table 1). Thus, the inhibitory generalization gradient was narrower than the excitatory one, indicating that the inhibitory effect of the CS− is more specific than the excitatory effect of the CS+.

## 4. Discussion

We investigated olfactory generalization in ants to determine how olfactory experience shapes their evaluation of odour similarity. We compared olfactory absolute and differential conditioning with a systematic analysis involving several odour combinations and showed for the first time, to our knowledge, that odour similarity depends on the way ants learned odourants. Compared with absolute conditioning, differential conditioning improved odour discrimination. Olfactory similarity relationships are, therefore, not immutable but vary with the conditioning procedure, i.e. how animals learn about the relationship between stimuli. Moreover, our modelling of behavioural performances indicates that the enhanced olfactory discrimination after differential learning relies on the interaction between excitatory and inhibitory generalization gradients mediated by the rewarded (CS+) and the punished odour (CS−), respectively.

Odour generalization in ants depended on structural similarity between aldehydes as shown by the response obtained after absolute conditioning: in this case, generalization was inversely related to the difference in the number of carbon atoms between the conditioned and the test odour, a phenomenon previously observed both in vertebrates [7,8,39] and invertebrates [11,12,40], including *C. aethiops* [14]. However, odour generalization (i.e. odour similarity) depended also on the procedure used to train the ants. After absolute conditioning, the ants' response was similar for the conditioning odour and for odours differing in one carbon atom; yet behavioural discrimination between these same odours was improved after differential conditioning. This result calls into question the absolute nature of olfactory perception as different similarity relationships between odours can be found depending on the conditioning procedure. Thus, studies on olfactory perception should carefully consider that the way a subject perceives odour similarity relationships depends on the way it learnt to respond to odours. Comparable conclusions have been reached for colour similarity relationships in bees, where absolute and differential conditioning yield different discrimination and generalization performances for the same colour stimuli [16,17,41]. It thus seems that perceptual performances, irrespectively of the modality considered, are significantly modulated by the kind of experience gathered by an animal.

In the insect brain, odours are first processed in the antennal lobes (AL), where olfactory receptor neurons located on the antennae synapse with local interneurons and projection neurons. The latter convey information to the Kenyon cells (KCs) of the mushroom bodies (MB), a structure involved in learning and memory [42] and to the lateral horn (LH) [43]. Extrinsic neurons (ENs) of the MBs (receiving input from KCs), and probably of the LH, provide output to premotor regions and are thought to be important for driving and organizing behavioural responses [44–47]. Electrophysiological and imaging studies have shown that similarity in neural activity patterns in the AL correlates with chemical similarity in terms of carbon-chain length (e.g. for aldehydes [12,32,48,49]) and with behavioural odour similarity [12,23,49]. Besides, changes in the neural signatures of odours as a result of olfactory learning have been found both in the AL and the MB [50–52]. For instance, differential conditioning decorrelates the neural representation of odours in both structures [23,50,53,54], thus facilitating response-pattern differentiation with training. Therefore, we expect that the experience-dependent changes in behavioural generalization and/or discrimination observed in our study translate into increased or decreased similarity in the ant's neural representation of odours.

In our study, the improved discrimination abilities displayed by the ants trained with differential conditioning were, however, not extended to all tested odours but were observed for odours that had a carbon-chain length relation to the CS+ similar to that of the CS−. Generalization gradients obtained after differential conditioning were asymmetric around the CS+; i.e. the level of response was maximal for the CS+ and higher levels of generalization were recorded for novel stimuli away from the CS+ in the direction opposite to the CS−; a phenomenon called ‘area shift’ [36], a less extreme version of the ‘peak shift effect’ [4,15,36,38]. Area shift (or peak shift) in olfactory generalization has already been observed in moths trained to discriminate alcohols differing in two carbons or alcohols and ketones differing in two carbons [13], and in honeybees trained to distinguish binary odour mixtures varying in the proportion of mixture components [53,55]. Therefore, this effect appears to be consistent in insects learning to discriminate similar odours.

Both the enhanced perceptual discrimination induced by discrimination learning and area shift (or peak shift) are commonly accounted for by assuming an additive interaction between excitatory and inhibitory generalization gradients mediated by the positively (CS+) and negatively (CS−) reinforced stimuli, respectively [4,15,38]. Learning to discriminate similar stimuli that differ along the same perceptual dimension induces excitatory and inhibitory generalization gradients, which overlap along this dimension, thus determining higher discrimination for stimuli lying between the CS+ and the CS−. For these stimuli, the algebraic sum of gradients results in near-to-zero response levels, which makes them well differentiable from the CS+. Our results show that area shifts in generalization gradients after differential conditioning were better accounted for by a multiplicative rather than by an additive interaction between excitatory and inhibitory generalization gradients. This can be understood if we interpret the excitatory and inhibitory generalization gradients as probability distributions of two interacting, independent processes. In this interpretation, the excitatory generalization gradient would represent the probability to be excited to respond to a stimulus (based on its similarity to a CS+) and the inhibitory generalization gradient would represent the probability to be inhibited from responding (based on the similarity to a CS−). The event of an animal responding is then the combination of the two events of being excited to respond and not being inhibited to respond, i.e. the probability to respond is the product of the probability to be excited multiplied by 1 minus the probability to be inhibited. A familiar example for this type of model, albeit from a different domain, is the phenomenological description of the opening of ion channels by Hodgkin & Huxley [56].

Our proposal converges with recent neuro-computational models proposed for honeybees to account for olfactory differential conditioning or peak shift [47,57,58]. These models assume that the population of ENs is divided into neurons inducing proboscis extension response (similar to MaLER) and neurons inducing proboscis retraction. The behavioural outcome is determined by mutual inhibition in the EN population that implements a ‘winner-takes-all’ mechanism between extension and retraction ENs. Learning occurs as a result of an alteration of the synaptic connections between KCs and ENs. Training with the CS+ increases the number or strength of excitatory connections between KCs that are active in response to the CS+ and ENs that activate proboscis extension, and decreases the connections with ENs that activate proboscis retraction. Conversely, training with the CS− increases connections with ENs that activate proboscis retraction and decreases connections with ENs that activate proboscis extension. As similar odours would activate similar KC populations [47], they would activate overlapping ENs populations. In these models, the probability of response to an odour relies on two independent probabilistic processes: the probability that extension ENs are excited to fire and the probability that retraction ENs are excited to fire. The overall behavioural outcome is determined by the competition, in the form of mutual inhibition, between extension and retraction ENs. These processes could relate with our findings: the excitatory and inhibitory generalization gradients would reflect the probability of response of extension and retraction ENs after conditioning, according to the olfactory similarity with the CS+ and with the CS−, respectively. The multiplicative interaction between the gradients would correspond to the competition between extension and retraction ENs. The finding that the multiplicative interaction of gradients provides a better account of the data lends some support to the idea of competing extension and retraction ENs over a model where excitatory and inhibitory inputs superimpose linearly onto premotor neurons for responding.

We found that inhibitory generalization gradients were narrower than excitatory ones, meaning that the inhibitory effect of the CS− is more specific than the excitatory effect of the CS+. This higher specificity would be suitable for learning the discrimination of the CS+ from the CS−. Indeed, an excessive overlap of excitatory and inhibitory generalization gradients would decrease considerably the ants' response to the CS+. The fact that the inhibitory generalization gradient affects all tested odours (including the CS+) despite its higher specificity, would explain why it was more difficult for ants to learn to discriminate similar stimuli than learning the absolute properties of the CS+ or why it is generally more difficult to learn to discriminate similar odours than dissimilar odours [10,28,59]. This hypothesis is supported by our results showing that learning to discriminate the odours was more difficult when the standard deviation of the inhibitory generalization gradient was high (i.e. heptanal+/nonanal− and octanal+/hexanal−) than when it was low (i.e. hexanal+/octanal− and nonanal+/heptanal−) (table 1 and figure 1). Studies in bees have shown that the shape of the generalization gradient obtained after differential conditioning (and in these cases, the magnitude of the peak shift) could vary according to some stimulus characteristics, such as the relative difference between the positive and the negative reinforcements or the relative frequency of CS+ and CS− trials [55,60]. This indicates that the width of excitatory and inhibitory gradients depends on these and probably other parameters [61]. For instance, in our case, a reinforcement more aversive than quinine or an increase of the number of CS− trials could have enlarged the width of the inhibitory generalization gradients, making them probably larger than the excitatory generalization gradients. Thus, different individual experiences could induce high or low levels of generalization to novel but similar stimuli, depending on the salience and abundance of the reinforced stimuli, and on the payoffs of responding to or ignoring them.

Our work indicates that a multiplicative interaction between two independent processes: the conditioned excitation and the conditioned inhibition mediated by the CS+ and the CS−, respectively, accounts for the improvement of olfactory discrimination in ants. Further experiments should investigate whether our model provides a better prediction of the generalization gradients obtained after differential conditioning in other taxa. Electrophysiological and optophysiological measurements of neural responses in the ants' olfactory circuit [31,32,62,63] may help understanding the neural basis of these processes and how and where along this circuit such interaction occurs.

## Supplementary Material

Manuscript Perez et al Proc R Soc Electronic Supplementary Material Final.docx

## Supplementary Material

Manuscript Perez et al Proc R Soc Raw Data.docx

## Data accessibility

The datasets supporting this article have been uploaded as part of the electronic supplementary material.
