# Supplementary material for: Olfactory experience shapes the evaluation of odour similarity in ants: a behavioural and computational analysis
Source: Proc Biol Sci. 2016 Aug 31;283(1837):20160551. doi: 10.1098/rspb.2016.0551 (PMC5013785; doi:10.1098/rspb.2016.0551)
Supplement: Manuscript Perez et al Proc R Soc Electronic Supplementary Material Final.docx [file rspb20160551supp1.docx]

**Electronic Supplementary Material**

**Olfactory experience shapes the evaluation of odour similarity in ants**

**Margot Perez^1, 2, 3^, Patrizia d’Ettorre^1, 2, 3^, Thomas Nowotny^4^ and Martin Giurfa^2, 3*^**

^1^ Laboratory of Experimental and Comparative Ethology (LEEC), University Paris 13, Sorbonne Paris Cité, Villetaneuse, France

^2^ Centre National de la Recherche Scientifique (CNRS), Research Centre on Animal Cognition (UMR5169), Toulouse, France

^3^ University Paul-Sabatier, Research Centre on Animal Cognition (UMR5169), Toulouse, France

^4^ Centre for Computational Neuroscience and Robotics, School of Engineering and Informatics, University of Sussex, Brighton, UK

*** Corresponding author:** [martin.giurfa@univ-tlse3.fr](mailto:martin.giurfa@univ-tlse3.fr)

1. **Study Organism**

Six queenright colonies of *Camponotus aethiops* were collected in May 2012 and April 2013 at Pompertuzat (Midi-Pyrénées, France, latitude 43.5°, longitude 1.516667°) and kept in the laboratory (25°C, light-dark cycle= 12:12, 60% humidity) in artificial nests composed of two plastic boxes connected by a plastic hose. One of the boxes was covered by cardboard and contained a plaster floor to form the nest; the other box, exposed to light, was the foraging arena. The inner sides of the two boxes were coated with Fluon® to prevent ants from escaping. Ants were fed twice a week with a diet of carbohydrates, proteins and vitamins [1]; water was provided *ad libitum*. Three weeks prior to the onset of the experiment, the ants’ diet was changed to *Tenebrio molitor* mealworms (protein) and water *ad libitum* but no carbohydrates were provided to increase the insects’ motivation for sucrose used as reward during conditioning.

1. **Conditioning and test procedures**

Ants were subjected to either absolute or differential conditioning of the *maxilla-labium* extension response (MaLER), an appetitive reaction to sucrose stimulation [2].

In differential conditioning, four odorant combinations, each one presenting two odours differing in two carbons, were trained: hexanal+/octanal-; heptanal+/nonanal-; octanal+/hexanal-; nonanal+/heptanal-, were “+” indicates the presence of reward and “-” that of punishment. Training consisted of 12 trials (6 CS+ and 6 CS- trials). Trials were presented in pseudo-random order, e.g. ABBABAABABBA, i.e. before each experiment, a new sequence of CS+ and CS- was determined in which none of the stimuli was given more than two consecutive times. In each group, the sequence started with a CS+ for half of the individuals; the contingency was inversed for the other half.

Each trial lasted 1min. Twenty-five seconds after placing the ant under a binocular, a CS was presented during 5s to the ant’s head by blowing an air puff with the syringe placed at 2cm. Three seconds after the onset of odour presentation the ant’s *maxilla-labium* was stimulated during 5s with either sucrose (eliciting the MaLER) in the CS+ trials, or with quinine in the CS- trials. Thus, the overlap between odour and reinforcement was always 2s. The ant was then left in the conditioning place during 27s in order to impede a predictive, forward association between context and reinforcement. An air extractor was placed behind the ant in order to remove remaining odour stimulations.

In absolute conditioning, four aldehydes were trained: hexanal+; heptanal+; octanal+; nonanal+. Training consisted of 6 CS+ trials of 1 min each, which followed the same procedure of the CS+ trials described for differential conditioning (see above). As absolute and differential conditioning differ in their number of trials (6 and 12, respectively), we equated the number of placements between both protocols by adding 6 blank trials in absolute conditioning. In each of these trials, ants were placed in the apparatus during 1min without any stimulation. Thus, ants were placed 12 times in total in the apparatus (6 CS+ trials and 6 blank trials in pseudo-random order).

For both conditioning procedures, the average inter-trial interval between two CS+ trials was 30 min. Individuals that did not respond at least four times to the sucrose reward were discarded (3.11% in total) to prevent confounding effects of a low motivation for the appetitive US on acquisition rates of the CS+.

In both cases, ants were tested with the four aldehydes in a randomized order fifteen minutes after the last conditioning trial. As for training, each test lasted 1min. Within each test, odours were presented during 5s without reinforcement. After the end of the test phase (all four odorants tested), a droplet of sucrose was presented to each ant to verify the presence of MaLER to the appetitive US. Ants that did not respond to that stimulation (1.04% in total) were not included in the statistical analyses as their low appetitive motivation could impact the level of responses to the trained and tested odours. Mortality rate during the experiments was of 2.67%.

1. **Data analysis**

All statistical analyses were performed with R-2.15.0 [3]. In training and test trials, the ant’s response was scored as 1 when MaLER was visible upon odour presentation; 0 otherwise. The percentage of ants responding to the odours was then calculated. Generalized linear mixed models (GLMM, package lme4; [4] with a binomial error structure (logit-link) were used to analyse the acquisition data. In all GLMMs, the ants’ response was used as response variable. Trials were coded as predictor variable (covariate), and individuals and colony of origin as random factors in order to allow for repeated measurements and adjust for colony origin. In the case of differential conditioning, the stimulus (CS+ and CS-) was also included as fixed factor. Interaction between trials and stimulus was included in the model to detect differences in slopes along the trials for the two types of CS. Variations in the response to the CS+ or the CS- along trials were revealed with post-hoc tests by applying the same GLMM without the factor stimulus to the respectively reduced set of data.

To test for the influence of the conditioning procedure on the rate of acquisition of the CS+, only the responses to the CS+ of ants trained with absolute or differential conditioning were used as response variable, and the conditioning procedure was also included as fixed factor. Interaction between trials and conditioning procedure was included in the model to detect differences in slopes for the CS+ along the trials between ants trained with the two conditioning procedures.

Differences in the ants' response to the CS+ and the CS- in the test after differential conditioning and differences in CS response between the test and the last conditioning trial after absolute and differential conditioning were detected by means of a McNemar’s Chi Square test. Differences in the response to CS+ in the test between ants subjected to absolute and differential conditioning were detected with a Fisher's Exact Test. The ants’ responses to the four aldehydes in the test were compared with Cochran’s Q tests and multiple McNemar’s Chi Square tests with sequential Bonferroni corrections were applied for pairwise comparisons between test odours.

1. **Generalization gradients modelling : choice of the model**

All fits were performed as a least-mean-squares fit with the *lsqnonlin* function in Matlab (Natick, MA). As stated in the main manuscript, we analysed the behavioural responses by fitting a simple model of Gaussian excitatory and inhibitory generalisation gradients for the CS+ and CS- odours respectively. The Gaussian function G_σ_ was fitted with maximal amplitude 100, standard deviation σ, and centred on the CS+ to the response percentages obtained after absolute conditioning and G_σ_ × (1- G_σ’_) was fitted to the data obtained after differential conditioning, with G_σ’_ being centred on CS- (see fit #1 below in table S1, and figure 3).

| # | p | | Residual errors of fit | | | | | | | |
| --- | --- | --- | --- | --- | --- | --- | --- | --- | --- | --- |
|  |  |  | hex+/oct- | oct+/hex- | hept+/non- | non+/hept- | mean | mean×#p | | |
| 1 | $\sigma, \sigma'$ | | 10.536 | 25.793 | 14.083 | 34.730 | 21.286 | 42.572 | | |
| 2 | $\sigma, \sigma'$ | | 53.626 | 48.835 | 38.474 | 56.227 | 49.291 | 98.582 | | |
| 3 | $\sigma, A, \mu, \sigma'$ | 9.268 | | 16.412 | 13.353 | 30.296 | 17.332 | 69.328 |  |  |
| 4 | $\sigma, \sigma^{'},A$ | 10.536 | | 20.439 | 14.084 | 33.464 | 19.631 | 58.892 | |  |

**Table S1:** **Residual errors of the four fitting procedures.** “#”: number of the fitting procedure; “p”: parameters included in the fit. Residual errors of fit are expressed as the square root of the sum of squared residuals; for fit #3 we used the additional penalty terms for negative response values; these are not included in the errors reported in this table. As in every fitting procedure there is a trade-off between the number of free parameters and the goodness of the fit; we therefore provide the product of mean residual error and number of parameters as an additional indication for the quality of each fitting procedure. The multiplicative fits are much better than the additive fits with respect to this criterion.

In the peak shift literature [5–8] a different, additive model is often employed, where the likelihood of response is expressed as the linear sum of an excitatory and an inhibitory gradient. We tried to fit our data with such a model initially but observed that with a direct two parameter fit, the results were quite unsatisfactory and had artefacts of negative response probabilities (fit #2 in table S1, figure S1). Indeed, experimentally, animals either respond or do not respond to the test stimulus, therefore generalization gradients values range from 0 to 100% of animals responding to the stimulus (i.e. always positive). When we introduced additional cost terms (C, see below) into the fit that strongly penalized negative response values and added two additional parameters (the mean µ and the amplitude A), we were able to achieve good fits (fit #3 in table S1, figure S2), but their interpretation was difficult: the obtained inhibitory generalisation gradient was not centred on the CS-; an observation that has no obvious interpretation.

$$C= \sum_{i=1}^{4} {(m_{i}-f_{\left\{ p \right\}}\left( x_{i} \right))}^{2}+k\sum_{i=0}^{5} {([f_{\left\{ p \right\}}\left( x_{i} \right)]\_)}^{2}$$

where $f_{\left\{ p \right\}}$ denotes the function fitted with parameters $\left\{ p \right\}$; $x_{i}$ denotes the difference in carbon-chain length between the CS+ and the test odour $i$; $m_{i}$ denotes the fraction of MaLER observed for the odors tested; $[f]\_$ denotes the negative part of $f$, i.e. $min\{f,0\}$; and $k$ is a free parameter for the weighting of the second cost term. In our case, we used $k=5$. We also introduced two additional points to the second term of the cost function to also avoid negative generalisation gradients further away from the CS+ and CS-. These points were $x_{0}=x_{1}-1$ and $x_{5}=x_{4}+1$. The effect of the second term of C was that if the curve of the generalization gradient obtained after differential conditioning was negative for the four odours used or for odours having one carbon less (i.e. pentanal) or one carbon more (i.e. decanal), then an extra cost proportional to the amount below zero was added.

Interestingly, when introducing an additional parameter (the amplitude A) into the multiplicative fit, we were also able to achieve good fits (fit #4 in table S1; figure S3). However, the relation between the goodness of fits and the number of parameters that were included in the fits was better for fit #1 than for fit #4 (see column “mean x p” in table S1).

In summary, the four presented fits were obtained by fitting the test responses after absolute conditioning with $100 \times e^{-\frac{\left( x-a \right)^{2}}{2\sigma^{2}}}$ and simultaneously, the test responses after differential conditioning with

Fit #1: the function $100 \times e^{-\frac{{(x-a)}^{2}}{2\sigma^{2}}} \times\left( 1-e^{-\frac{({x-b)}^{2}}{2\sigma^{'2}}} \right)$

Fit #2: the function $100 \times\left( e^{-\frac{{(x-a)}^{2}}{2\sigma^{2}}}-e^{-\frac{{(x-b)}^{2}}{2\sigma^{'2}}} \right)$

Fit #3: the function $100 \times\left( e^{-\frac{{(x-a)}^{2}}{2\sigma^{2}}}{-A e}^{-\frac{{(x-b-\mu)}^{2}}{2\sigma^{'2}}} \right)$

Fit #4: the function $100 \times e^{-\frac{{(x-a)}^{2}}{2\sigma^{2}}} \times\left( 1-{A e}^{-\frac{({x-b)}^{2}}{2\sigma^{'2}}} \right)$

Here, a and b denote the carbon chain length of the CS+ and CS- respectively.

We also initially tried other functional fits, including simple exponential decay, and polynomial decay with varying numbers of parameters (data not shown). However, the Gaussian fits performed best, which is in accordance with previous studies (see [8]).


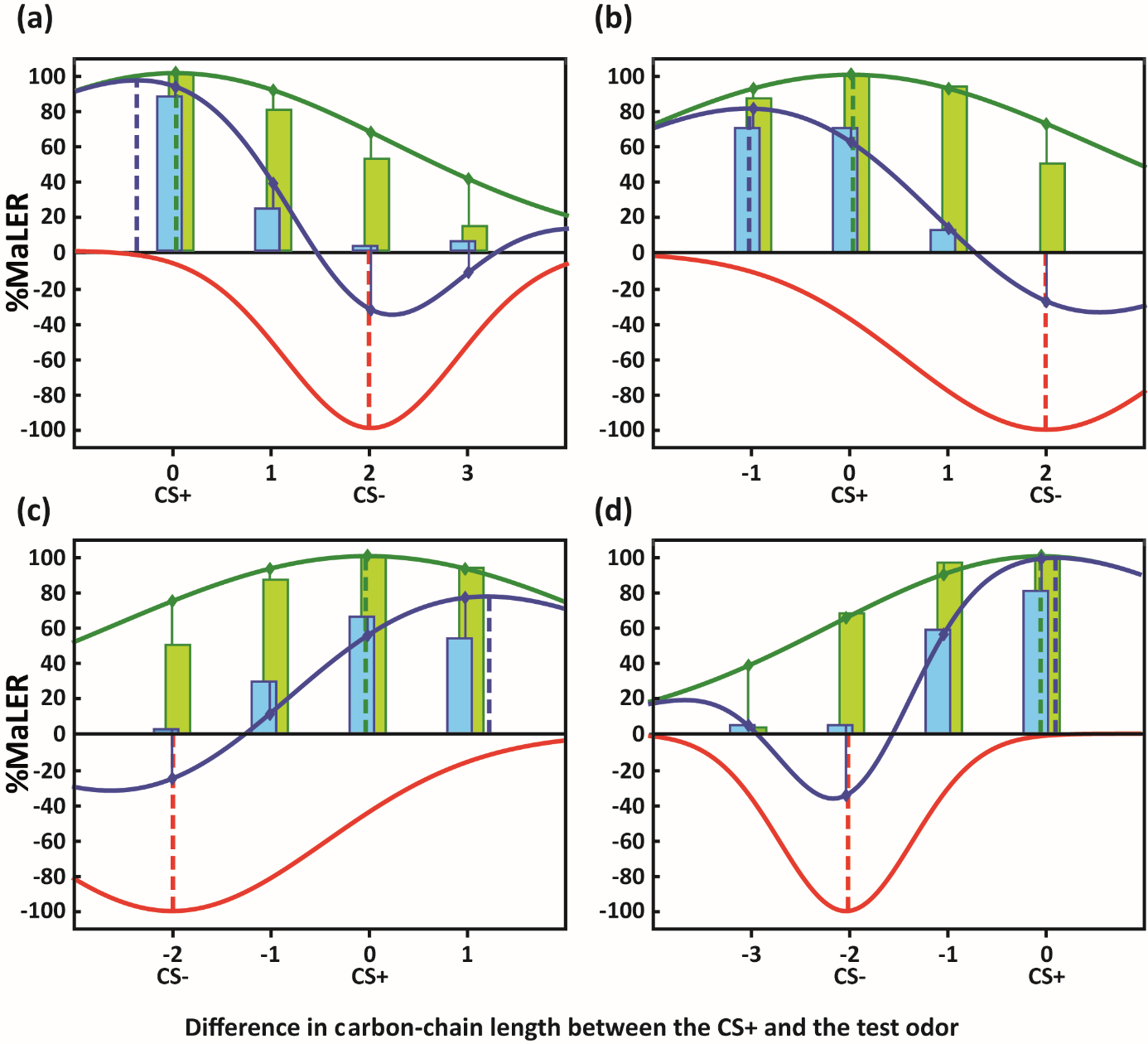


**Figure S1:** **Least mean square fits of the experimental data with the sum of a positive (excitatory) and a negative (inhibitory) Gaussian generalization gradient with two free parameters (fit #2).** Green and blue bars are response percentages obtained in the behavioural experiments after absolute conditioning and differential conditioning, respectively. Green lines (excitatory gradients) are the fits to the response percentages after absolute conditioning and the blue lines the fits to the responses after differential conditioning. The red lines show the hypothesized inhibitory generalization gradient that, when combined with the green lines produce the blue lines. **(a)** hexanal+ vs. hexanal+/octanal-; **(b)** heptanal+ vs. heptanal+/nonanal-; **(c)** octanal+ vs. octanal+/hexanal-; **(d)** nonanal+ vs. nonanal+/heptanal-. Note the regions of negative values appearing in all the blue lines.


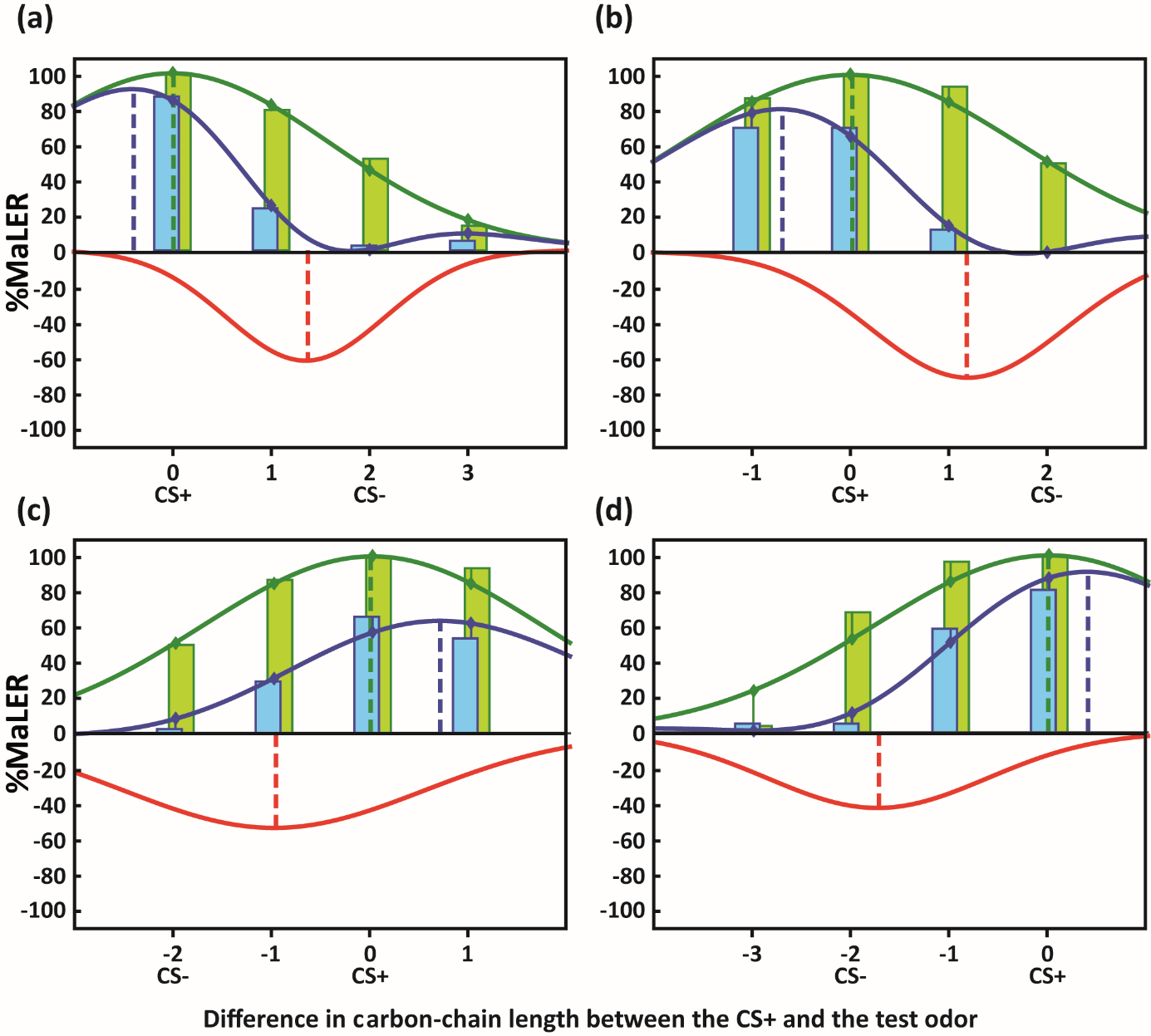


**Figure S2: Least mean square fits of the experimental data with the sum of a positive (excitatory) and a negative (inhibitory) Gaussian generalization gradient with four free parameters and additional imposed penalties for negative response values (fit #3).** Colour code as in Figure S1. **(a)** hexanal+ vs. hexanal+/octanal-; **(b)** heptanal+ vs. heptanal+/nonanal-; **(c)** octanal+ vs. octanal+/hexanal-; **(d)** nonanal+ vs. nonanal+/heptanal-. The fit is of high quality but less good than fit #1 when taking into account the number of free parameters. The position of the peaks of the inhibitory gradients is difficult to interpret and 4 free parameters for 8 data points appears excessive.


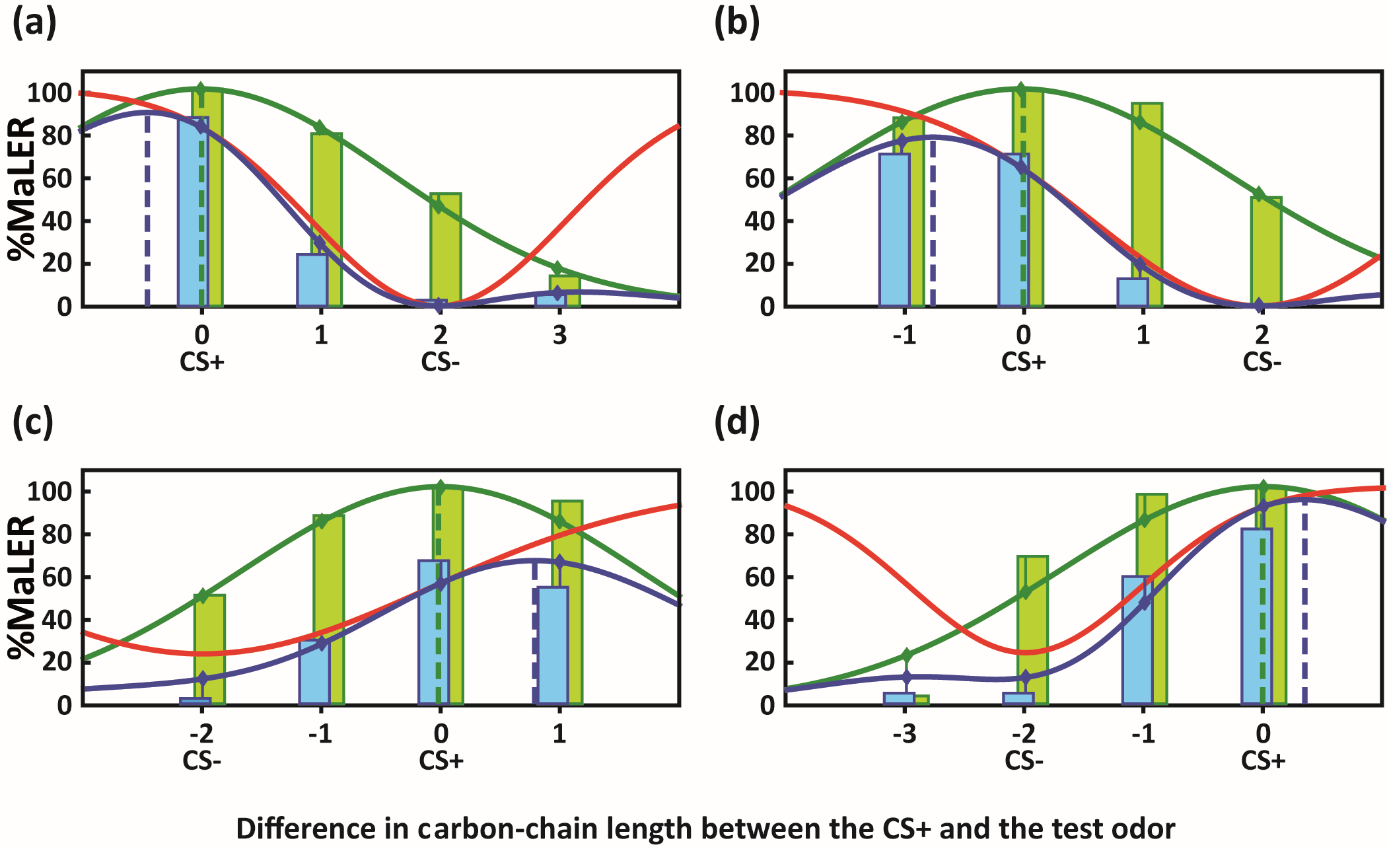


**Figure S3: Least mean square fits of the experimental data with the product of a positive (excitatory) and a negative (inhibitory) Gaussian generalization gradient with three free parameters (fit #4).** Colour code as in Figure S1. **(a)** hexanal+ vs. hexanal+/octanal-; **(b)** heptanal+ vs. heptanal+/nonanal-; **(c)** octanal+ vs. octanal+/hexanal-; **(d)** nonanal+ vs. nonanal+/heptanal-.

**References**

1. Bhatkar, A. & Whitcomb, W. H. 1970 Artificial diet for rearing various species of ants. *Fla. Entomol.* **53**, 229. (doi:10.2307/3493193)

2. Guerrieri, F. J. & d’Ettorre, P. 2010 Associative learning in ants: conditioning of the maxilla-labium extension response in *Camponotus aethiops*. *J. Insect Physiol.* **56**, 88–92. (doi:10.1016/j.jinsphys.2009.09.007)

3. {R Development Core Team} 2011 *R: A Language and Environment for Statistical Computing*. Vienna, Austria: R Foundation for Statistical Computing.

4. Bates, D., Maechler, M. & Bolker, B. 2011 *lme4: Linear mixed-effects models using S4 classes*.

5. Spence, W. K. 1936 The nature of discrimination learning in animals. *Psychol. Rev.* **43**, 427–449. (doi:10.1037/h0056975)

6. Spence, K. W. 1937 The differential response in animals to stimuli varying within a single dimension. *Psychol. Rev.* **44**, 430–444. (doi:10.1037/h0062885)

7. Hanson, H. M. 1959 Effects of discrimination training on stimulus generalization. *J. Exp. Psychol.* **58**, 321–334. (doi:10.1037/h0042606)

8. Ghirlanda, S. & Enquist, M. 2003 A century of generalization. *Anim. Behav.* **66**, 15–36. (doi:10.1006/anbe.2003.2174)
