# Supplementary material for: Olfactory experience shapes the evaluation of odour similarity in ants: a behavioural and computational analysis
Source: Proc Biol Sci. 2016 Aug 31;283(1837):20160551. doi: 10.1098/rspb.2016.0551 (PMC5013785; doi:10.1098/rspb.2016.0551)
Supplement: Manuscript Perez et al Proc R Soc Raw Data.docx [file rspb20160551supp2.docx]

**Supporting data**

Eight groups of ants were trained with four aldehydes following either absolute (hexanal+; heptanal+; octanal+; nonanal+) or differential conditioning (hexanal+/octanal-; heptanal+/nonanal-; octanal+/hexanal-; nonanal+/heptanal-) of the MaLER. All groups of ants have been then tested with the four aldehydes 15 min after training.

## hexanal+

## heptanal+

## octanal+

## nonanal+

## hexanal+/octanal-

## heptanal+/nonanal-

## octanal+/hexanal-

## nonanal+/heptanal-
